# Accuracy of robotic arm-assisted versus computed tomography-based navigation in total hip arthroplasty using the direct anterior approach: a retrospective study

**DOI:** 10.1186/s12891-024-07891-3

**Published:** 2024-10-04

**Authors:** Tomoya Okazaki, Takashi Imagama, Yuta Matsuki, Hiroshi Tanaka, Eiichi Shiigi, Takehiro Kaneoka, Takehiro Kawakami, Kazuhiro Yamazaki, Takashi Sakai

**Affiliations:** 1 Department of Orthopedic Surgery, Yamaguchi University Graduate School of Medicine, Ube, Yamaguchi 755-8505 Japan; 2 Department of Orthopedic Surgery, Yamaguchi Prefectural Grand Medical Center, Hofu, Yamaguchi 747-8511 Japan

**Keywords:** Total hip arthroplasty, Direct anterior approach, Robotic surgical procedures, Surgical navigation systems, computer-assisted surgery

## Abstract

**Background:**

A robotic arm-assisted and a computed tomography (CT)- based navigation system have been reported to improve the accuracy of component positioning in total hip arthroplasty (THA). However, no study has compared robotic arm-assisted THA (rTHA) to CT-based navigated THA (nTHA) concerning accuracy of cup placement and acetabular fractures using the direct anterior approach (DAA). This study aimed to compare the accuracy of cup placement and the presence of intraoperative acetabular fractures between rTHA and nTHA using DAA in the supine position.

**Methods:**

We retrospectively investigated 209 hips of 188 patients who underwent rTHA or nTHA using DAA (rTHA using the Mako system: 85 hips of 79 patients; nTHA: 124 hips of 109 patients). After propensity score matching for age and sex, each group consisted of 73 hips. We evaluated clinical and radiographic outcomes, comparing postoperative cup orientation and position, measured using a three-dimensional templating software, to preoperative CT planning. Additionally, we investigated the prevalence of occult acetabular fracture.

**Results:**

Clinical outcomes were not significantly different between the groups at 1 year postoperatively. The mean absolute error of cup orientation was significantly smaller in the rTHA group than in nTHA (inclination: 1.4° ± 1.2° vs. 2.7° ± 2.2°, respectively; *p* = 0.0001, anteversion: 1.5° ± 1.3° vs. 2.2° ± 1.7°, respectively; *p* = 0.007). The cases within an absolute error of 5 degrees in both RI and RA were significantly higher in the rTHA (97.3%) than in nTHA group (82.2%) (*p* = 0.003). The absolute error of the cup position was not significantly different between the two groups. The prevalence of occult acetabular fracture did not differ significantly between the two groups (rTHA: *n* = 0 [0%] vs. nTHA: *n* = 1 [1.4%]).

**Conclusion:**

Cup placement using DAA in the supine position in rTHA was more accurate with fewer outliers compared to nTHA. Therefore, rTHA performed via DAA in a supine position would be useful for accurate cup placement.

## Introduction

Primary total hip arthroplasty (THA) is a useful surgery with long term results for reducing pain and improving hip function [1]. However, complications such as dislocation, implant impingement, periprosthetic fracture, and infection, can occur [2, 3]. In particular, dislocation is one of the main reasons for revision THA [2]. Mispositioning of the acetabular cup is one risk factor for dislocation [4, 5]; therefore, optimal and accurate cup placement is essential for preventing implant impingement during THA.

Technologies such as robotic-assisted surgery, computed tomography (CT)-based navigation, imageless navigation, and accelerometer navigation have been reported to increase the accuracy and precision of acetabular cup placement [6–10]. In particular, robotic arm-assisted THA with the Mako system (Mako; Stryker, Kalamazoo, MI, USA) and CT-based navigation systems are capable of creating patient-specific models from preoperative CT images and can adjust the cup orientation and position during surgery [11]. Thus, both technologies enable the reproduction of preoperative plans for cup orientation and positioning [6, 12–14]. The Mako system is comprised of a CT-based navigation system and a robotic arm with haptic control of the instruments. Previous reports have shown that robotic arm-assisted THA achieved more accurate cup placement than THA using manual guidance and fluoroscopy [13–16]. However, only a few reports have compared robotic arm-assisted surgery with CT-based navigated THA in terms of cup placement [6, 17, 18]. According to clinical outcomes, previous reports have compared robotic arm-assisted surgery with manual or portable navigation; however, to the best of our knowledge, no studies have compared it with CT-based navigated THA [19, 20]. Moreover, in previous studies, differences in the surgical approach affected cup orientation with and without the Mako systems [21, 22]. To our knowledge, no reports have compared robotic arm-assisted THA (rTHA) to CT-based navigated THA (nTHA) using the direct anterior approach (DAA) in the supine position.

Recently, the press-fit technique for cementless cups has become a popular fixation technique [23]. Hasegawa et al. showed the prevalence rate of periprosthetic occult fractures of the acetabulum, which were not found on routine postoperative radiographs, to be 8.4% [24]. Intraoperative acetabular fractures most frequently occur during insertion of the acetabular component [25, 26]. In the rTHA, the surgeon can confirm the cup’s center of rotation (COR) on display during cup insertion. In contrast, in the nTHA, the surgeon can confirm the cup position with respect to final reamer position during cup insertion. Because the surgeon can confirm whether the component reaches the target position, the surgeon might avoid further cup impaction after full cup seating. It may lead to reduce fracture risk. To our knowledge, no reports compared the prevalence rates of occult fractures between rTHA and nTHA using DAA.

We hypothesized that rTHA would offer better cup placement accuracy than nTHA and have similar short-term clinical outcomes. This study aimed to clarify the accuracy of cup orientation and positioning and to compare the prevalence rates of occult acetabular fractures between the rTHA and nTHA groups with matched patient background analysis.

## Methods

### Patients

**Fig. 1:**
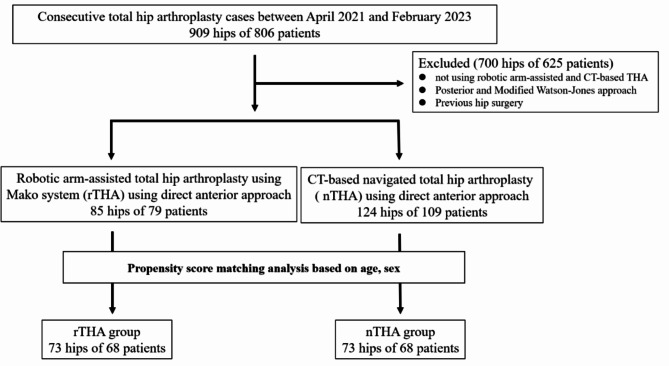
Patient demographic flow chart

Between April 2021 and February 2023, we examined 909 hips of 806 consecutive THA patients. Inclusion criteria were: (1) males and females, (2) age 18–90 years, (3) undergoing elective primary hip arthroplasty using rTHA or nTHA. Exclusion criteria were: (1) not using robotic arm-assisted and CT-based navigation, (2) posterior and modified Watson-Jones approach, (3) previous hip surgery. Based on these criteria, we assessed 85 hips of 79 patients who underwent rTHA using the Mako system (Stryker Kalamazoo, MI, USA) and 124 hips of 109 patients who underwent nTHA using a CT-based navigation system (Stryker CT-Hip system Ver1.3; Stryker Kalamazoo, MI, USA). This study was approved by our institutional review board (H2020-068). All patients provided informed consent. Propensity score matching was used to match the patients’ backgrounds for sex and age between the two groups using the JMP^®^ program (version 16.0; SAS Institute Inc., Cary, NC, USA). Finally, 73 hips from each group were included in this study (Fig. 1). One facility performed rTHA and the other nTHA. The surgeons overlapped in both groups. Regarding patient demographics, no significant differences were found between the two groups (Table 1).

**Table 1:** Patients’ demographics

	rTHA (*n* = 73 hips)	nTHA (*n* = 73 hips)	*p*-value
Number of patients	68	68	
Sex (patient)			0.48
male	10	9	
female	58	59	
Age (years)	69.3 ± 8.8	69.2 ± 8.6	0.98
BMI (kg/m^2^)	24.2 ± 4.4	25.4 ± 4.7	0.094
Diagnosis (hip)			0.25
DDH	39	32	
OA	24	27	
ONFH	6	7	
RDC	4	2	
SIF	0	4	
RA	0	1	
Cup size (hip) (mm)			0.17
44 mm	0	2	
46 mm	6	6	
48 mm	21	36	
50 mm	24	13	
52 mm	10	6	
54 mm	7	6	
56 mm	4	3	
58 mm	1	1	

Values are expressed as means ± standard deviation or as numbers (n). p-values in bold indicate statistical significance (*p* < 0.05)

*rTHA* Robotic arm-assisted total hip arthroplasty, *nTHA* Navigated total hip arthroplasty using computed tomography-based navigation system, *BMI* Body mass index, *DDH* Developmental dysplasia of the hip, *OA* Osteoarthritis; *ONFH* Osteonecrosis of the femoral head, *RDC* Rapidly destructive coxarthropathy, *SIF*, Subchondral insufficient fracture of the femoral head, *RA* Rheumatoid arthritis

### Preoperative planning

All preoperative plans were made using CT-based simulation software ZedHip (LEXI, Tokyo, Japan) based on preoperative CT images obtained with a helical CT scanner (Aquilion Precision System, Toshiba Medical System, Tokyo, Japan) (SOMATOM go, Siemens Healthcare, Erlangen, Germany). The slice thickness and pitch were 1 mm in both groups. The functional pelvic plane (FPP) was used to plan the cup orientation. The target cup orientation basically aimed for a radiographic inclination (RI) of 40° and radiographic anteversion (RA) of 15°. The RA target was set in consideration of the risk of iliopsoas impingement caused by anterior cup edge overhang. Subsequently, the preoperative plans made by ZedHip were traced to each software in either the Mako system or CT-based navigation system.

### Surgical technique

All surgeries were performed using DAA in the supine position on a normal operating table under general anesthesia. The skin incision was made 1 cm distal and lateral to the anterior superior iliac spine, parallel to the tensor fasciae latae. Dissection through the interval between tensor fasciae latae and sartorius muscles was performed. We incised the anterior capsule as a triangular flap, based on the femoral attachment, along the side of the vertical band of the iliofemoral ligament [27]. After anterior capsulotomy, femoral neck osteotomy was performed following preoperative planning. After implantation, the capsule was repaired. One team performed all surgeries under the supervision of two senior hip arthroplasty surgeons with > 20 years of experience (I.T. and T.H.). All hips were implanted with cementless hemispherical cups. The acetabular component was placed using press-fit fixation. Screw fixation for cup implantation was performed when the surgeon deemed it necessary.

### rTHA

**Fig. 2:**
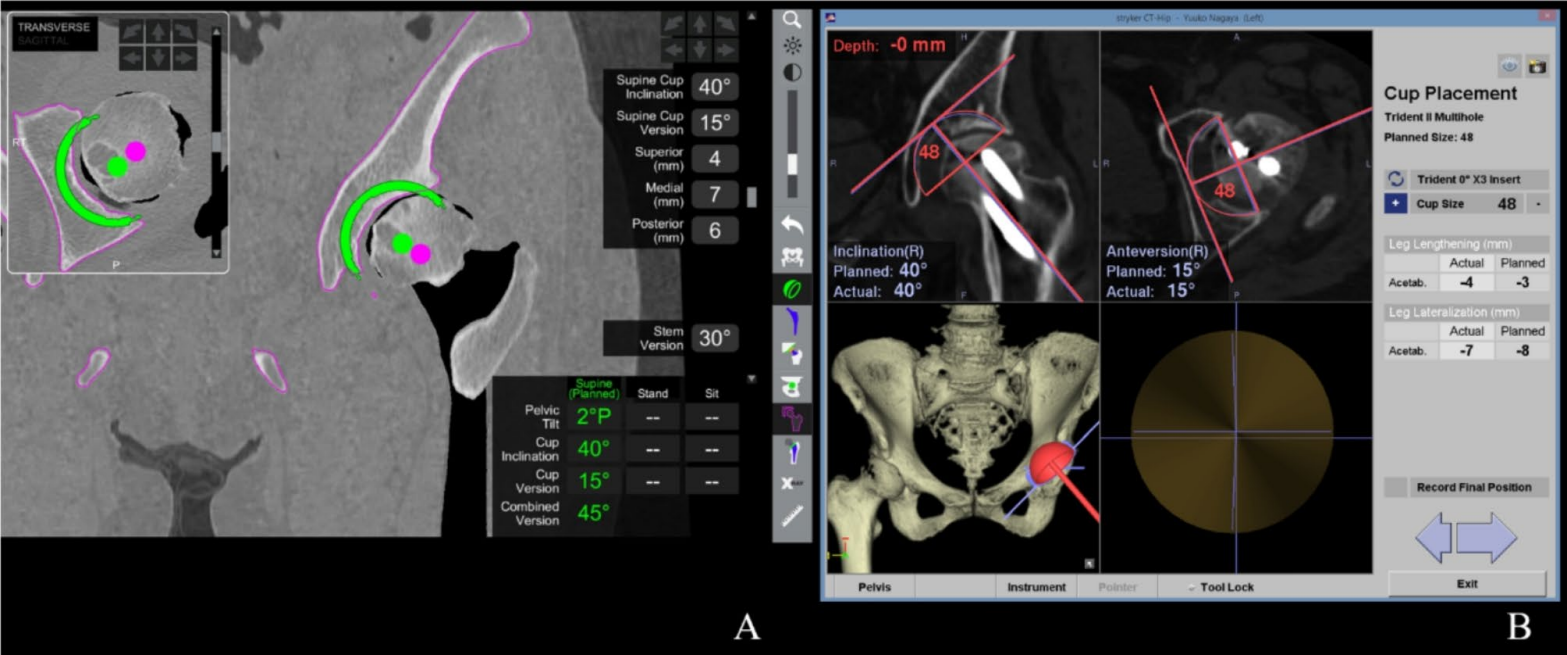
Acetabular component orientation and positioning **(A)** Robotic arm-assisted system and **(B)** CT-based navigation system

In the rTHA group, three pins (4-mm diameter) were inserted at the contralateral side of the iliac crest to attach to the pelvic array. The surface registration area were the external iliac plate, anterior periarticular area within 5 cm of the acetabular rim, and intra-acetabular region. After pelvic registration, the acetabulum was prepared with a planned reamer using a robotic arm. Acetabular cup implantation was based on the orientation and positioning indicated by the software, which was displayed as the real-time error of inclination, anteversion, and COR (Fig. 2A). All acetabular cups were Trident HA hemispherical cup (Stryker, Kalamazoo, MI, USA).

### nTHA

In the nTHA group, two pins (5-mm diameter) were inserted at the contralateral side of the iliac crest to attach to the pelvic array. The surface registration area was an external iliac plate and anterior periarticular area within 5 cm of the acetabular rim. An acceptable range for the surface match registration accuracy was 1 mm. The acetabulum was reamed using real-time errors of inclination and anteversion. Finally, the cup was implanted in accordance with inclination, anteversion, and COR (Fig. 2B). The acetabular components were PINNACLE (DePuy Synthes, Warsaw, IN, USA) in 38 hips, Trident II Tritanium (Stryker) in 26 hips, Trident HA hemispherical (Stryker) in 5 hips, and Anasta cup (Teijin Nakashima Medical, Okayama, Japan) in 4 hips.

### Evaluations

Clinical outcomes were assessed using the following measures: pre- and postoperative Japanese Orthopedic Association (JOA) scores as physician-reported outcomes, the Japanese Orthopedic Association Hip Disease Evaluation Questionnaire (JHEQ) score, and the postoperative Forgotten Joint Score-12 (FJS-12) as a patient-reported outcome (PROMs) [28–30]. JOA scores ranged from 0 (worst) to 100 (best) and JHEQ scores ranged from 0 (worst) to 84 (best). Additionally, we examined the operative time, intraoperative blood loss, perioperative complications, and complications up to 1 year postoperatively.

Radiographic outcomes were assessed cup orientation and positioning. We routinely obtained CT images at two weeks postoperatively to assess for complications such as occult fractures. The data were uploaded to the three-dimensional templating software, ZedHip. The ZedHip automatically matched the preoperative and postoperative FPP and differences between the pre-and postoperative orientation and cup center position were measured.

**Fig. 3:**
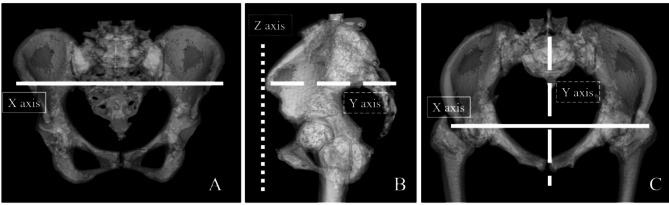
Cup position is defined in three-dimensional space with the X-axis, Y-axis, and Z- axis. The straight line indicates the X-axis **(A)**. The X-axis (horizontal axis) is the line connecting the bilateral anterior superior iliac spines. A straight line with short dots represents the Z-axis **(B)**. The Z-axis (vertical axis) was vertical to the X-axis. A straight line with long dots represents the Y-axis (**B**, **C**). The Y-axis (sagittal axis) was perpendicular to the X- and Z-axis

The absolute error of the cup orientation (RI and RA) between preoperative planning and postoperative CT measurements were investigated. Each value was compared between the two groups. We assessed outliers of RI and RA that were > 5 degrees. Additionally, cup orientation with and without screws in rTHA and nTHA were assessed. The absolute error of the COR between the preoperative planning and postoperative CT measurements were also investigated. The COR was defined by the coordinates on the x-, y-, and z-axes using ZedHip. The x-axis (horizontal axis) is the line connecting the bilateral anterior superior iliac spines. The z-axis (vertical axis) was vertical to the x-axis, parallel to the FPP, and through the pubic tubercle. The y-axis (sagittal axis) was perpendicular to the x- and z-axes (Fig. 3). Each value was compared between the two groups. Additionally, we examined the cup orientation and positioning based on BMI categories (below 25, 25 to 30, and above 30), Kellgren-Lawrence grade (KL grade) of osteoarthritis, and the presence or absence of screw insertion within each group. The prevalence rates of acetabulum occult fracture using postoperative CT images were compared between the two groups.

### Statistical analysis

Continuous variable data are presented as the mean ± standard deviation. We used non-parametric tests because the distributions of the preoperative JOA and JHEQ score significantly deviated from normality. Continuous and categorized data were analyzed using the Mann–Whitney *U* and chi-squared tests, respectively. All statistical analyses were performed using JMP version 16. Differences were considered statistically significant at a p-value of 0.05. A power analysis using G*power (3.1; Düsseldorf, Germany) indicated that the sample size of this study (effect size f, 0.50; alpha error probability, 0.05; power, 0.90) was 70 hips. Thus, 73 hips in each group would be sufficient to detect statistically significant differences.

## Results

### Clinical outcomes

There were no significant differences in the pre- and postoperative JOA scores, JHEQ scores, and FJS-12 between the two groups (Table 2). Surgical time and blood loss were not significantly different between the two groups (surgical time: rTHA 107.7 ± 25.4 min, nTHA 110.6 ± 24.7 min, *p* = 0.34; blood loss: rTHA 281.6 ± 182.3 ml, nTHA 326.6 ± 2 10.1 ml, *p* = 0.12). No cup-related complications occurred in either group. Perioperatively, one case of periprosthetic fracture around the femoral stem was observed in the rTHA group, and two cases of superficial infection were observed in the nTHA group. No complications were observed in either group for up to 1 year postoperatively.

**Table 2:** Comparison of clinical outcomes between the two groups

Clinical outcomes	rTHA	nTHA	*p*-value
Preoperative			
JOA score	46.8 ± 12.4	48.6 ± 11.6	0.12
JHEQ score	22.2 ± 11.8	18.1 ± 13.1	0.064
Postoperative			
JOA score	87.9 ± 8.9	90.8 ± 9.4	0.089
JHEQ score	59.4 ± 14.2	61.9 ± 16.5	0.26
FJS-12	71.5 ± 20.9	68.7 ± 23.2	0.70

Values are expressed as mean ± standard deviation. P-values in bold indicate statistical significance (*p* < 0.05)

*rTHA* Robotic arm-assisted total hip arthroplasty, *nTHA* Navigated total hip arthroplasty using CT-based navigation system, *JOA score* Japanese Orthopaedic Association score, *JHEQ* Japanese Orthopaedic Association Hip-Disease Evaluation Questionnaire, *FJS-12* Forgotten joint score-12

### Radiographic outcomes

**Fig. 4:**
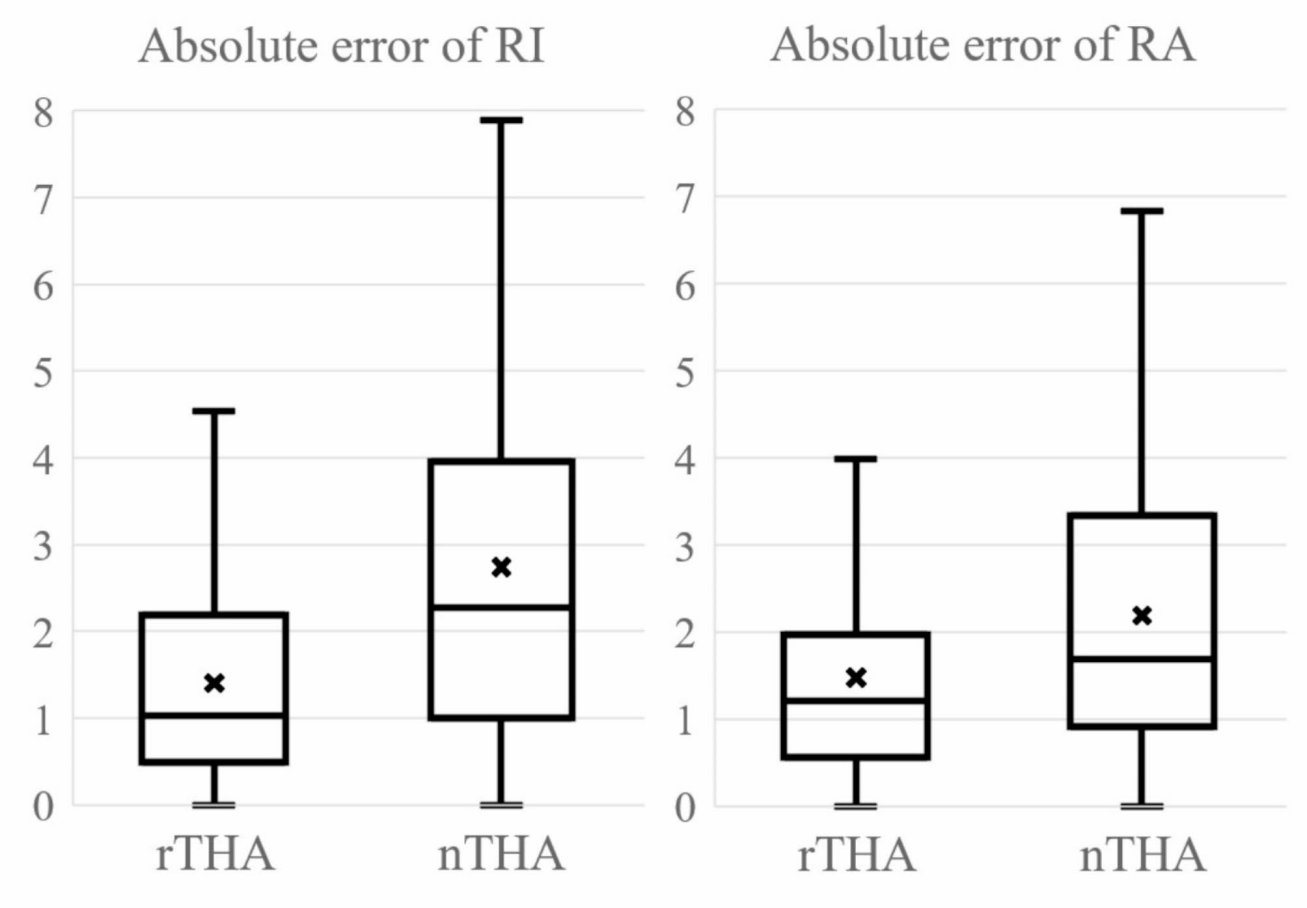
The absolute error of radiographic inclination and anteversion between two groups

**Fig. 5:**
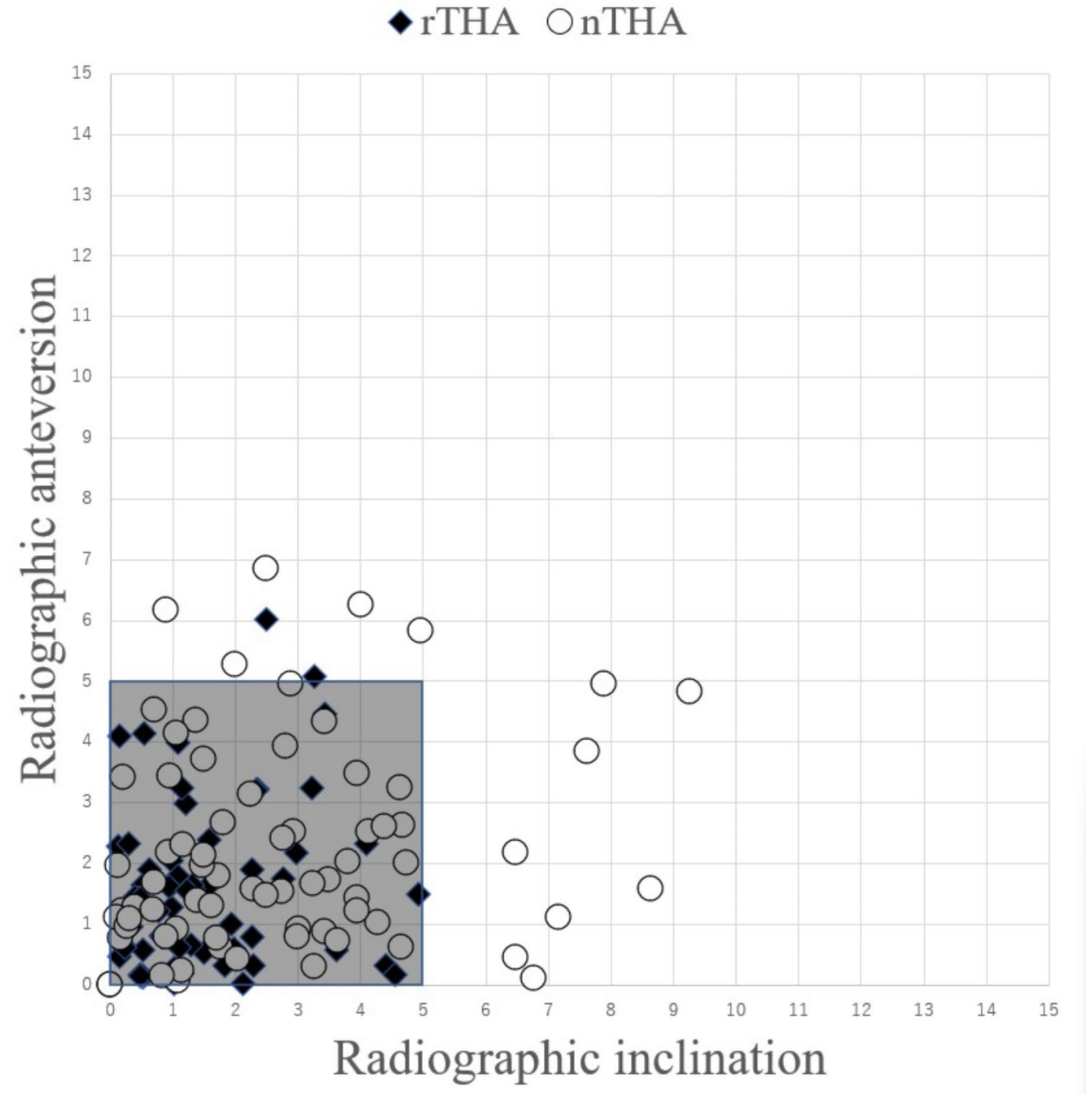
Scattergram of postoperative cup orientation plots with the absolute error of inclination and anteversion in the rTHA and nTHA groups

**Fig. 6:**
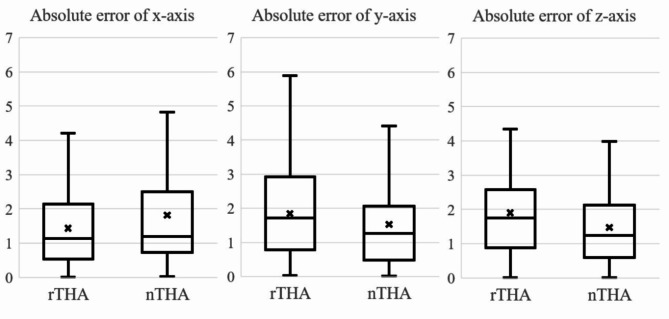
The absolute error of cup center position (x-, y-, and z-axes) in the rTHA and the nTHA groups

The absolute error of RI in the rTHA group was significantly smaller than that in the nTHA groups (1.4° ± 1.2°, 2.7° ± 2.2°, respectively) (*p* = 0.0001). Similarly, the absolute error of RA in the rTHA group was smaller than that in the nTHA group (1.5 °± 1.3°, 2.2° ± 1.7°, respectively) (*p* = 0.007) (Fig. 4). Scatter plot of the absolute error of RI and RA are shown in Fig. 5. The rates of placement within 5 degrees were 97.3% (71 hips) in the rTHA group and 82.2% (60 hips) in nTHA; there was significant difference between the two groups (*p* = 0.002). In the rTHA group, the absolute errors of RI were 1.4° ± 0.6° with screw (*n* = 4) and 1.4° ± 1.2° without screw (*n* = 69) and 1.0° ± 1.5° with screw and 1.5° ± 1.3° without screw in the absolute errors of RA. In the nTHA group, the absolute errors of RI were 2.2° ± 2.0° with screw (*n* = 22) and 2.9° ± 2.3° without screw (*n* = 51) and 1.8° ± 1.6° with screw and 2.3° ± 1.7° without screw in the absolute errors of RA. Statistical analysis was not performed due to the small number of cases, but results were similar in both groups for the presence or absence of screws. The mean absolute errors of the x-, y-, and z-axes were 1.4 ± 1.2 mm, 1.8 ± 1.3 mm, and 1.8 ± 1.2 mm in the rTHA group, respectively, and 1.8 ± 1.6 mm, 1.5 ± 1.3 mm, and 1.5 ± 1.2 mm in the nTHA group, respectively. There was no significant difference between two groups (Fig. 6).

There was no statistically significant difference in cup orientation or position between groups based on KL grade and the presence/absence of screw insertion (Tables 3 and 4). Regarding BMI, there was no significant difference in cup orientation. Additionally, higher BMI was not associated with greater deviation in cup placement (Table 5).

**Table 3:** Comparison of cup orientation and position according to KL grade variations between the two groups

	rTHA			nTHA		
	KL grade 3 (*n* = 9)	KL grade 4 (*n* = 53)	*p*-value	KL grade 3 (*n* = 8)	KL grade 4 (*n* = 52)	*p*-value
RI (°)	0.9° ± 0.9°	1.6° ± 1.3°	0.22	2.9° ± 1.8°	2.7° ± 2.0°	0.95
RA (°)	1.8° ± 2.0°	1.5° ± 1.2°	0.94	2.7° ± 1.5°	2.1° ± 1.7°	0.12
X axis (horizontal) (mm)	1.1 ± 1.3	1.5 ± 1.2	0.47	1.5 ± 1.2	1.8 ± 1.7	0.97
Y axis (sagittal) (mm)	2.0 ± 1.6	1.8 ± 1.2	0.99	1.2 ± 1.1	1.7 ± 1.3	0.71
Z axis (vertical) (mm)	1.4 ± 1.2	2.0 ± 1.3	0.25	1.5 ± 0.9	1.5 ± 1.1	0.82

*rTHA* Robotic arm-assisted total hip arthroplasty, *nTHA* Navigated total hip arthroplasty using CT-based navigation system, *KL* Kellgren-Lawrence classification, *RI* Radiographic inclination, *RA* Radiographic anteversion

**Table 4:** Comparison of the cup orientation and position with and without screws between the two groups

	rTHA			nTHA		
	with screw (*n* = 4)	without screw (*n* = 69)	*p*-value	with screw (*n* = 22)	without screw (*n* = 51)	*p*-value
RI (°)	1.4° ± 0.6°	1.4° ± 1.2°	0.91	2.2° ± 2.0°	2.9° ± 2.3°	0.49
RA (°)	1.0° ± 1.5°	1.5° ± 1.3°	0.67	1.8° ± 1.6°	2.3° ± 1.7°	0.54
X axis (horizontal) (mm)	2.5 ± 1.9	1.4 ± 1.1	0.56	2.2 ± 2.1	1.5 ± 1.3	0.69
Y axis (sagittal) (mm)	1.4 ± 1.4	1.9 ± 1.3	0.95	1.3 ± 1.2	1.6 ± 1.3	0.62
Z axis (vertical) (mm)	1.5 ± 1.3	1.9 ± 1.3	0.52	1.2 ± 1.1	1.6 ± 1.2	0.20

*rTHA* Robotic arm-assisted total hip arthroplasty, *nTHA* Navigated total hip arthroplasty using CT-based navigation system, *RI* Radiographic inclination, *RA* Radiographic anteversion

**Table 5:** Comparison of cup orientation and position according to BMI in each group

BMI	rTHA				nTHA			
	BMI < 25 (*n* = 49)	25 ≤ BMI ≤ 30 (*n* = 18)	30 < BMI (*n* = 6)	*p*-value	BMI < 25 (*n* = 39)	25 ≤ BMI ≤ 30 (*n* = 22)	30 < BMI (*n* = 12)	*p*-value
RI (°)	1.4° ± 1.2°	1.7° ± 1.4°	0.7° ± 0.7°	0.14	2.7° ± 2.1°	2.8° ± 2.4°	2.8° ± 2.6°	0.99
RA (°)	1.6° ± 1.4°	1.5° ± 1.1°	1.0° ± 0.6°	0.81	2.3° ± 1.9°	1.7° ± 1.1°	2.7° ± 1.7°	0.30
X axis (horizontal) (mm)	1.1 ± 0.9	2.3 ± 1.4	1.6 ± 1.6	**0.020**	1.7 ± 1.8	2.0 ± 1.5	1.9 ± 1.3	0.46
Y axis (sagittal) (mm)	1.9 ± 1.4	1.7 ± 1.1	1.8 ± 1.4	0.86	1.3 ± 1.2	1.8 ± 1.4	1.9 ± 1.3	0.13
Z axis (vertical) (mm)	1.9 ± 1.2	2.1 ± 1.7	1.4 ± 1.1	0.49	1.3 ± 1.1	1.7 ± 1.2	1.5 ± 1.3	0.28

Values are expressed as mean ± standard deviation. P-values in bold indicate statistical significance (*p* < 0.05)

*BMI* Body mass index, *THA* Total hip arthroplasty, *CT* Computed tomography, *RI* Radiographic inclination, *RA* Radiographic anteversion, *rTHA* Robotic arm-assisted total hip arthroplasty, *nTHA* Navigated total hip arthroplasty using CT-based navigation system,

There were no acetabular fractures intraoperatively in either group. No cases of occult fracture of the acetabulum occurred in the rTHA group and one case of occult fracture was found in the nTHA group. There was no significant difference between the two groups. In case of occult fracture, additional treatment and limited weight-bearing were unnecessary.

## Discussion

The rTHA group was more accurate for cup orientation in both RI and RA than the nTHA group using DAA in the supine position. The number of cases within 5 degrees of the absolute error for both RI and RA were significantly greater in the rTHA group than nTHA. There were no significant differences in the clinical outcomes, cup position, and the prevalence rate of occult fracture between the two groups. This study is the first report to show the differences in cup placement between rTHA and nTHA using DAA in the supine position. In this study, the absolute errors of RI and RA were significantly smaller in the rTHA group than in the nTHA group. Additionally, the rates of outlier cup placement were significantly smaller in the rTHA group than in nTHA.Although some reports assessed cup orientation using robotic arm-assisted THA, there have been no reports accurately evaluating the use of CT after rTHA through DAA in the supine position (Table 6) [6, 12–14, 16–18, 31–33]. Moreover, no reports have compared rTHA to nTHA using DAA cup orientation (Table 7) [6, 17, 18]. We consider the reasons for this difference between the two groups, such as the number of pins for connecting the tracker to pelvis, surface registration area, and use of a hand-held or robotic arm. First, tracker stability can affect surgical accuracy. In the external fixation of the pelvis for pelvic fracture, the number and diameter of pins have been reported to affect frame rigidity [34]. Thus, the tracker stability was also considered to depend on the number and diameter of pins. The rTHA group had three pins (4-mm diameter) compared to two pins (5-mm diameter) in the nTHA group. The rTHA group had more pin insertions, however, they were of a smaller diameter than those used in the nTHA group. Direct comparison between the two groups is difficult because the number of pins and diameters are different in the two groups, however, there were no cases of obvious intraoperative pin loosening in both groups. Second, the surface registration areas for rTHA and nTHA were different in this study. In the rTHA group, the registration areas were the external iliac plate and anterior periarticular area within 5 cm of the acetabular rim, and the intra-acetabular region following the instruction of the Mako system. In contrast, only the external iliac plate and anterior periarticular area within 5 cm of the acetabular rim were used as the registration areas in the nTHA group, according to a previous report [35]. Docquier et al. showed that a larger sampling area increased registration accuracy [36]. Thus, registration area differences may affect registration accuracy, which may affect cup orientation. Third, In the nTHA group, the surgeon determined the reaming direction using a handheld reamer [6]. In contrast, in the rTHA group, the haptic arm placed the reamer within 15° of the cup placement target from center. Previous reports have shown that RI decreases while RA increases in the nTHA group preoperative plan because of the difference in interface stress between the craniomedial and inferior portion in the acetabulum [37, 38]. In the rTHA, the robot haptic arm guides the cup holder to the target cup orientation; however, in the nTHA group, cup placement is performed handheld. Therefore, in nTHA, surgeons need to control the cup orientation by the surgeon himself. In rTHA, the surgeon does not need to control the cup orientation. The use of haptic arm is an advantage of rTHA in preventing cup malalignment.

**Table 6:** The accuracy of robotic arm-assisted THA in various surgical approaches

Author	Approach	Number of hips	Assessment modality	RI	RA	Target angle	
						RI	RA
Shaw et al. [12]	PL	141	radiograph	42.5 ± 5.3	25.6 ± 5.4	NA	22–25
Guo et al. [14]	PL	45	CT	41.5 ± 4.2	21.1 ± 5.7	40	20
Kamara et al. [16]	PL	98	radiograph	40.5 ± 3.7	19.4 ± 4.4	40	20
Shibanuma et al. [17]	PL	30	radiograph	42.2 ± 2.2	20.3 ± 1.6	40	20
Redmond et al. [31]	PL	35	radiograph	39.9 ± 2.5	17.4 ± 3.4	40	20
Kanawade et al. [32]	PL	43	CT	39.1 ± 3.8	18.9 ± 4.1	40	20
Ando et al. [6]	PL/mWJ	27/2	CT	2.0 ± 1.4*	1.9 ± 1.4*	40	15
Sato et al. [33]	mWJ	84	CT	1.1 ± 1.0*	1.2 ± 1.1*	40	-†
Tamaki et al. [18]	ALS	52	CT	1.1 ± 0.9*	1.3 ± 1.0*	38.8 ± 1.5	15.8 ± 1.7
Domb et al. [13]	PL/DAA	52/14	radiograph	40.9 ± 3.2	18.4 ± 3.7	40	20
Our study	DAA	73	CT	1.4 ± 1.2*	1.5 ± 1.3*	40	15 or 20

Values are expressed as mean ± standard deviation

* Absolute error of the cup angle between the preoperative and postoperative measurement

† The cup anteversion angle was fine-tuned for each patient

Abbreviations: RI, Radiographic inclination; RA, Radiographic anteversion; PL, Posterolateral approach; NA, Not available; CT, Computed tomography; mWJ, Modified Watson-Jones approach; ALS, Anterolateral approach in the supine position; DAA, Direct anterior approach

**Table 7:** Direct comparison of radiographic inclination and anteversion between robotic arm-assisted THA and CT navigated THA

Author	Approach	Each number of hips	Assessment modality	RI				RA			
				rTHA	nTHA	Target angle (rTHA/nTHA)	*p*-value	rTHA	nTHA	Target angle (rTHA/nTHA)	*p*-value
Ando et al. [6]	PL/mWJ	27/2	CT	2.0 ± 1.4*	3.5 ± 2.4*	40/40	**0.007**	1.9 ± 1.4*	2.8 ± 2.3*	15/15	0.108
Shibanuma et al. [17]	PL	30	radiograph	42.2 ± 2.2	40.5 ± 4.5	40/40	0.07	20.3 ± 1.6	19.9 ± 3.6	20/20	0.11
Tamaki et al. [18]	ALS	52	CT	1.1 ± 0.9*	2.2 ± 1.5*	38.8/38.4	**< 0.01**	1.3 ± 1.0*	3.3 ± 2.5*	15.8/12.0	**< 0.01**
Our study	DAA	73	CT	1.4 ± 1.2*	2.7 ± 2.2*	40/40	**0.0001**	1.5 ± 1.3*	2.2 ± 1.7*	15 or 20/15 or 20	**0.007**

Values are expressed as mean ± standard deviation. P-values in bold indicate statistical significance (*p* < 0.05)

*Absolute error of the cup angle between the preoperative and postoperative measurement is indicated

*THA* Total hip arthroplasty, *CT* Computed tomography, *RI* Radiographic inclination, *RA* Radiographic anteversion, *rTHA* Robotic arm-assisted total hip arthroplasty, *nTHA* Navigated total hip arthroplasty using CT-based navigation system, *PL* Posterolateral approach, *mWJ* Modified Watson-Jones approach, *ALS* Anterolateral approach in the supine position, *DAA* Direct anterior approach

In our study, cup orientation regardless of the presence or absence of screws did not differ significantly between the two groups. Fujishiro et al. showed that the cup orientation could undergo changes during screw fixation [39]. Garcia reported the cases of either the lateralized cup position tended to use the screws [23]. If cup installation could not be done as planned, we considered that surgeons were more likely to perform screw insertion to improve stability. Tabata et al. reported that intraoperative primary cup stability was influenced by the exactness of the reaming procedure and accurate cup insertion [40]. We believe that there was no significant difference in the presence or absence of screws because rTHA and nTHA allows for accurate cup orientation and placement. It should be noted that the number of screw insertion cases in this study was small. A large-scale study will be necessary in the future.

In this study, COR levels were not significantly different between the two groups. No reports have compared the COR of rTHA to nTHA using DAA (Table 8) [6, 8, 18, 33, 41]. Compared to imageless navigation, rTHA and nTHA are both capable of creating a patient-specific model generated from preoperative imaging and intraoperative registration [11]. We considered that both technologies would be useful for the accuracy of cup COR.

In the present study, accurate placement of the cup in the supine position was enabled in the rTHA group using DAA. In DAA, previous reports used robotic arm-assisted surgery, navigation systems, and fluoroscopy (Table 9) [7–10, 42–45]. Use of a mechanical alignment guide and fluoroscopy tended to cause large absolute errors in the RA compared to nTHA using DAA [8, 45]. Compared to rTHA and nTHA, imageless navigation requires less equipment, spares exposing the patient to radiation, and removes the expense of preoperative imaging [11]. However, portable navigation cannot assess the cup position during surgery and create patient-specific models; there is also a risk of inaccuracies, such as pelvic deformity.

**Table 8:** Comparison of cup COR between robotic arm-assisted THA and navigated THA using CT-based navigation system

Author	Approach	Each number of hips	Assessment modality	rTHA			nTHA		
				X axis (horizontal) (mm)	Y axis (sagittal) (mm)	Z axis (vertical) (mm)	X axis (horizontal) (mm)	Y axis (sagittal) (mm)	Z axis (vertical) (mm)
Ando et al. [6]	PL/mWJ	27/2	CT	1.6 ± 1.6	1.8 ± 1.7*	2.2 ± 1.4†	1.9 ± 1.6	3.1 ± 2.5*	3.7 ± 2.5†
Tamaki et al. [18]	ALS	52	CT	1.3 ± 1.3	1.7 ± 1.5	2.0 ± 2.0	1.6 ± 1.4	1.8 ± 1.3	2.6 ± 2.3
Sato et al. [33]	mWJ	84	CT	1.2 ± 0.9	1.1 ± 0.9	2.0 ± 1.3	-	-	-
Matsuki et al. [8]	DAA	50	CT	-	-	-	2.1 ± 1.7	1.7 ± 1.4	1.8 ± 1.4
Iwana et al. [41]	PL	117	CT	-	-	-	1.9 ± 1.5	1.4 ± 1.2	1.9 ± 1.3
Our study	DAA	73	CT	1.4 ± 1.2	1.8 ± 1.3	1.8 ± 1.2	1.8 ± 1.6	1.5 ± 1.3	1.5 ± 1.2

Values are expressed as mean ± standard deviation. Absolute error of the cup center of rotation between the preoperative and postoperative measurement are indicated

Abbreviation: COR, Center of rotation; THA, Total hip arthroplasty; CT, Computed tomography; rTHA, Robotic arm-assisted total hip arthroplasty; nTHA, Navigated total hip arthroplasty using CT-based navigation system; PL, Posterolateral approach; mWJ, Modified Watson-Jones approach; ALS, Anterolateral approach in the supine position; DAA, Direct anterior approach

*Significant difference between the two groups

†Significant difference between the two groups

**Table 9:** The accuracy of radiographic inclination and anteversion using DAA

Author	Approach	Number of hips	Device	Assessment modality	RI	RA	Target angle	
							RI	RA
Kamath et al. [7]	DAA	33	CT free robotic assisted (ROSA)	radiograph	1.8 ± 1.3*	2.6 ± 2.3*	40	15
Matsuki et al. [8]	DAA	50	CT-based navigation (Stryker)	CT	2.8 ± 2.5*	2.8 ± 1.9*	40	15
Nogler et al. [42]	DAA	22	CT-based navigation (Stryker)	CT	1.3 (0.6–2.2) *	2.4 (1.0-3.2) *	45	20
Tsukada et al. [9]	DAA	69	Imageless-navigation (OrthoPilot)	CT	2.8 ± 2.5*	4.2 ± 3.0*	45	15
Lass et al. [43]	DAA	65	Imageless-navigation (Navitrack)	CT	3.0 ± 2.5*	5.5 ± 3.6*	40	15
Hasegawa et al. [10]	DAA	55	Accelerometer-based navigation (Naviswiss)	CT	4.1 ± 3.2*	4.3 ± 3.2*	40	15
Okamoto et al. [44]	DAA	115	Accelerometer-based navigation (Hip-align)	CT	3.1 ± 2.2*	2.8 ± 2.3*	40	15 or 20
Kolodychuk et al. [45]	DAA	99	Accelerometer-based navigation (Hip-align)	radiograph	1.8 ± 1.6*	3.2 ± 3.1*	40	15
Our study	DAA	73	Robotic arm-assisted (Mako)	CT	1.4 ± 1.2*	1.5 ± 1.3*	40	15 or 20

Values are expressed as mean ± standard deviation and parentheses indicted interquartile range

* Absolute error of the cup angle between the preoperative and postoperative measurement

*DAA* Direct anterior approach, *RI* Radiographic inclination, *RA* Radiographic anteversion, *CT* Computed tomography

In this study, we compared rTHA with nTHA, and found no differences in physician- and patient-reported outcomes in the short term. A previous study reported that postoperative FJS score tended to be higher in the rTHA group compared to the manual THA group, while no significant differences were found in other PROMs between the two groups [21]. Zahaf reported that semi-elliptical cracks tend to concentrate stress, which could lead to implant loosening [46]; however, robotic haptic arm by enabling more precise reaming, may reduce this risk and positively impact long-term outcomes. Although there was no significant difference in short-term PROMs, further research on long-term outcomes is necessary.

Our study showed that robotic surgery enabled more accurate cup placement than nTHA. However, not all hospitals can afford robotic systems owing to their high cost. Some studies suggest rTHA is cost-effective, citing reduced use of rehabilitation services [47] and a lower dislocation rate than manual THA [48]. Additionally, previous reports indicate that the COVID-19 pandemic limited surgical experience for orthopedic residents [49], and robotic systems could enhance training by offering more precise surgeries, further highlighting the potential cost-effectiveness of rTHA.

In the present study, occult fractures were detected in one hip in the nTHA group and no hips in the rTHA group. Hasegawa et al. showed that the prevalence rate of occult fractures of the acetabulum was 8.4%, and peripheral self-locking cups may increase this risk [25]. In this study, because only hemispherical cups were used, the acetabular cup design may not have affected these differences. The incidence of occult fracture was no significant difference in the two groups.

This study had limitations. First, the sample size is relatively small; however, it is reasonable to perform statistical analyses because the power analysis determined the sample size to be sufficient. In addition, the patient characteristics were matched using propensity score matching. Second, we did not investigate the clinical outcomes; we compared just acetabular cup orientation and position. Although these differences are statistically significant, it is unclear if there is a clinical difference. However, there is a significant difference in the outliers between the two groups. These differences may affect clinical outcomes. In the future, long-term follow-ups and clinical outcome evaluations will be necessary.

## Conclusions

The rTHA group exhibited a greater accuracy in cup orientation and fewer outliers than the nTHA group; however, the COR levels and prevalence of occult fractures were not significantly different between the two groups. The application of DAA rTHA could be useful for achieving more accurate cup placements with fewer outliers.

## Data Availability

Data associated with this study is retained at the Department of Orthopedic surgery, Yamaguchi university graduate school of medicine and Yamaguchi prefectural grand medical center. The datasets generated and/or analyzed in this study are available from the corresponding author on reasonable request. If there are any questions, please contact the corresponding author.

## Abbreviations

THA: Total hip arthroplasty
rTHA: Robotic arm-assisted total hip arthroplasty
nTHA: Navigated total hip arthroplasty using computed tomography-based navigation system
DAA: Direct anterior approach
PL: Posterolateral
COR: Center of rotation
FPP: Functional pelvic plane
RI: Radiographic inclination
RA: Radiographic anteversion
